# The pattern of skin diseases in the Qassim region of Saudi Arabia: What the primary care physician should know

**DOI:** 10.4103/0256-4947.72263

**Published:** 2010

**Authors:** Hani A. Al Shobaili

**Affiliations:** From the Department of Dermatology, College of Medicine, Qassim University, Saudi Arabia

## Abstract

**BACKGROUND AND OBJECTIVES:**

Epidemiological studies to determine the burden of skin diseases are important for proper health care planning. The purpose of this study was to find the pattern of skin diseases in our patients attending university-affiliated dermatologic clinics in the Qassim region.

**METHODS:**

We conducted a prospective study of all Saudi patients attending the Qassim University Medical College-affiliated dermatology clinics of the Ministry of Health for a period of 12 months from 1 March 2008 to 28 February 2009.

**RESULTS:**

The study included 3051 patients comprising 1786 (58.5%) males and 1265 (41.5%) females. Males outnumbered females (*P*<.05) (male-to-female ratio, 1.4:1). The mean age (standard error of the mean) of the patients was 25.3 (0.27) years. About 71% of the patients were between 5 and 34 years of age. The top five skin diseases were eczema/ dermatitis (19.5%), viral infections (16.6%), pilosebaceous disorders (14.4%), pigmentary lesions (11.2%) and hair disorders (7.6%). The major disorder in males was viral skin infections (20.0%), while eczema/dermatitis (20.7%) constituted the most prevalent skin disease in females. Seasonal variations were recorded in cases of pigmentary lesions, papulosquamous disorders and protozoal infections.

**CONCLUSION:**

Infectious skin diseases, eczema/dermatitis, pilosebaceous disorders, pigmentary lesions and hair disorders ranked as the top five skin diseases. Appropriate training programs for diagnosing and managing common skin diseases should be initiated for primary health care physicians and other general practitioners so as to decrease referrals to dermatology clinics.

Skin diseases are among the common skin problems that are encountered by primary care physicians. The prevalence of skin diseases in any region or country depends on various factors, such as genetics, racial constitution, social and hygienic standards, customs, nutritional status and climatic conditions. In addition, the diagnostic competence of doctors, expertise of dermatologists and availability of the latest diagnostic facilities play a crucial role.^1^

Every individual suffers from skin disease at some point in time during his lifetime. Conditions such as warts and acne are almost universal at certain ages.^2^ However, whether people recognize or report these common conditions varies according to the area affected and the severity of the problem. Epidemiological surveys to determine the burden of dermatological disorders in an area are important. The information gathered about the prevalence and spectrum of specific disorders helps in the development of strategies for proper health care planning. Such information also helps to establish proper preventive and research programs according to the need of the community.

The literature on the patterns of general and specific skin diseases is scanty, and only a few published reports are available on Saudi Arabia.^3^ Though community-based studies are the best to determine the incidence of a particular disease, they are difficult to carry out. As such, most of the studies to determine the incidence or prevalence of dermatological diseases are based upon hospital attendees.^4^,^5^

The Qassim region of Saudi Arabia is undergoing rapid development and urbanization, which may be affecting the spectrum of skin diseases in the area. No epidemiological survey is available to estimate the prevalence rate of various skin diseases in the area. The purpose of this study was to find out the pattern of skin diseases in our patients attending the university-affiliated dermatologic clinics in the Qassim region so that appropriate training programs on common skin diseases could be initiated for primary health care physicians and other general practitioners.

## METHODS

This prospective study was conducted in the Qassim University Medical College-affiliated dermatology clinics of the Ministry of Health, Qassim region, for a period of 12 months from 1 March 2008 to 28 February 2009. The Qassim region is located in the central part of the Arabian Peninsula, with a population of 1.08 million. It has hot and dry summers, cold winters and rainfall in April and May. The region has an agricultural belt with a sizeable farming community. Approval for conducting this study was obtained from the research and ethical committee of Qassim College of Medicine. Only Saudi patients who were permanent and original residents of the Qassim region were included in the study. Patients who were non-Saudis or of other ethnicities or expatriates were excluded from the study to avoid any confounding factors. All new and old patients attending a dermatology clinic for skin problems during this period were included in this study. All patients were seen by a consultant dermatologist. The diagnoses were mainly based on clinical examination, but were supported by laboratory investigations and histopathology wherever needed. Clinical data on age, sex and diagnosis were collected. All data were processed and analyzed using the Statistical Package for Social Sciences (SPSS version 14). Statistical significance was determined by the (2 and t tests. The level of significance was set at *P*<.05.

## RESULTS

**Figure 1:**
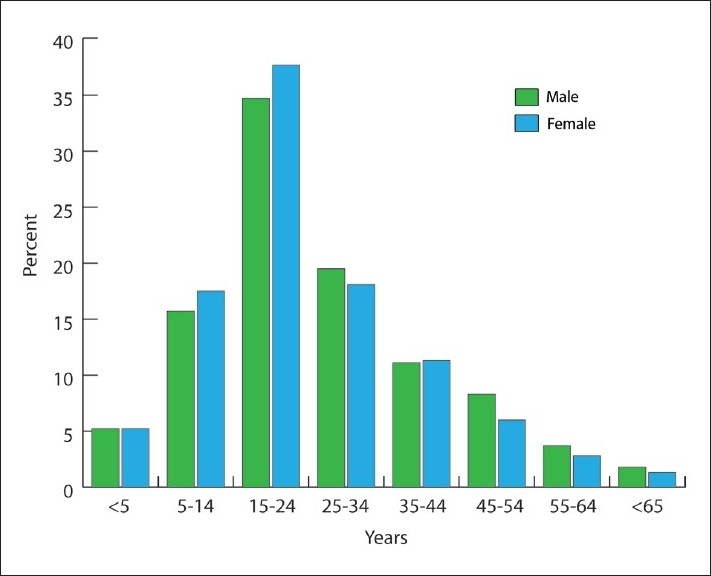
Patient distribution by age and sex.

Of 3051 patients with skin diseases seen during the study period, there were 1786 (58.5%) males and 1265 (41.5%) females. The male-to-female ratio was 1.4:1, and the difference was statistically significant (*P*<.05). The mean (SD) age of our patients was 25.3 (14.9) years. A statistically significant difference was seen in the mean (SD) age of males (26.0 [10.1]) as compared to that of females (24.4 [13.9]; *P*=.001). The distribution of males and females into various age groups is shown in Figure 1. Female patients outnumbered male patients younger than the age 24 years, but after that the number of male patients outgrew that of the female patients. Figure 1 also shows a clustering of patients at younger ages. About 71% of our patients were between 5 and 34 years of age, with a maximum number of patients presenting between ages 15 and 24 years (37.7% females and 34.7% males).

**Figure 2:**
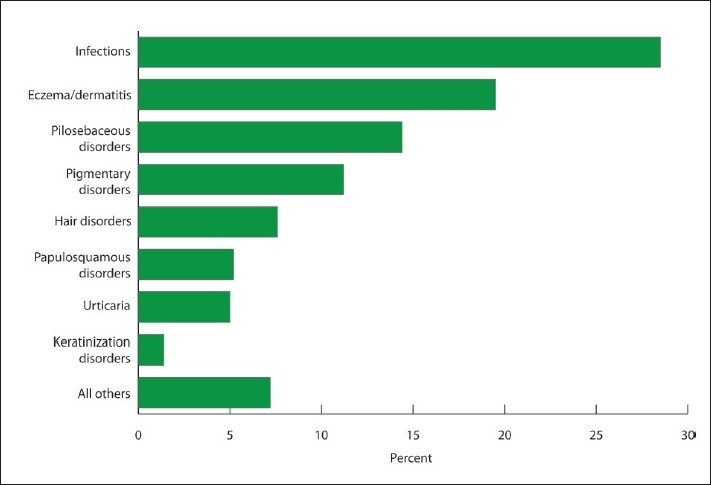
Overall ranking of skin disease.

Figure 2 shows the frequencies of various skin disorders in order of ranking. Of the total percentage of eczema/dermatitis cases (19.5%), atopic dermatitis and housewife dermatitis constituted 7.4% and 4.3% of those cases, respectively (**Table 1**). Of patients with viral infections (16.6%), viral warts were found in 13.2% of the viral infection cases. Among the pilosebaceous disorders (14.4%), acne vulgaris (13.5%) was the predominant condition. Among pigmentary disorders (11.2%), vitiligo, post-inflammatory hyperpigmentation and melasma were present in 5.2%, 3.7% and 1.9% of the patients, respectively. Diffuse hair loss and alopecia areata were present in 3.9% and 2.7% of the patients, respectively. Infections of all types accounted for 28.5% of skin diseases.

**Table 1:** Comparison of disease pattern between genders (n= 3051).

Disease	Male (n=1786)	Female (n=1265)	*P*
Eczema/dermatitis	333 (18.6)	262 (20.7)	.145
Viral infections	358 (20.0)	149 (11.8)	.001
Pilosebaceous disorders	210 (11.8)	230 (18.2)	.001
Pigmentary disorders	149 (8.3)	192 (15.2)	.001
Hair disorders	104 (5.8)	127 (10.0)	.001
Papulosquamous disorders	119 (6.7)	39 (3.1)	.001
Urticaria	83 (4.6)	71 (5.6)	.203
Fungal infections	99 (5.5)	52 (4.1)	.083
Bacterial infections	83 (4.6)	37 (2.9)	.019
Protozoal infection	88 (4.9)	4 (0.3)	.001
Keratinization disorders	24 (1.3)	18 (1.4)	.749
All others	136 (7.6)	84 (6.6)	.267

Values are number (percent).

The prevalence of different skin diseases differed between males and females. In males, viral infections were the most prevalent skin disorder, while in females eczema/dermatitis were the most prevalent. The prevalence rates of viral infections, papulosquamous disorders, bacterial infections and protozoal infection were significantly higher in males as compared to females, while prevalence rates of pilosebaceous disorders, pigmentary disorders and hair disorders were significantly higher in females as compared to males.

The spectrum of diseases varied in different age groups (**Table 2**). The eczema/dermatitis were predominant in the following age groups: <5 years (54.4%), 5-14 years (23.9%), 45-54 years (21.9%) and 55 years and above (30.5%). Viral skin infections were the most prevalent disorder affecting age groups 5-14 years (23.5%), 25-34 years (16.6%) and 35-44 years (19.3%). The predominant skin problem at 15-24 years of age was pilosebaceous disorders (29.3%). The prevalence of pigmentary disorders was significantly lesser in children under 5 years (3.8%) and older patients of age 55 years and above (6%). The prevalence of urticaria, on the other hand, was significantly higher in the older age group of 55 years and above (17.2%).

**Table 2:** Distribution of skin diseases by age groups (n= 3051).

		Age groups						
Disease	Total	<5 years (n=158) No. (%)	5-14 years (n=502) No. (%)	15-24 years (n=1097) No. (%)	25-34 years (n=577) No. (%)	35-44 years (n=342) No. (%)	45-54 years (n=224) No. (%)	55+ years (n=151) No. (%)
Eczema/dermatitis	595	86 (54.4)	120 (23.9)	152 (13.9)	84 (14.6)	58 (17.0)	49 (21.9)	46 (30.5)
Viral infection	507	15 (9.5)	118 (23.5)	172 (15.7)	96 (16.6)	66 (19.3)	24 (10.7)	16 (10.6)
Pilosebaceous disorders	440	2 (1.3)	25 (5.0)	321 (29.3)	70 (12.1)	9 (2.6)	9 (4.0)	4 (2.6)
Pigmentary disorders	341	6 (3.8)	60 (12.0)	134 (12.2)	58 (10.1)	43 (12.6)	31 (13.8)	9 (6.0)
Hair disorders	231	6 (3.8)	32 (6.4)	91 (8.3)	66 (11.4)	25 (7.3)	9 (4.0)	2 (1.3)
Papulosquamous disorders	158	7 (4.4)	41 (8.2)	40 (3.6)	27 (4.7)	16 (4.7)	16 (7.1)	11 (7.3)
Urticaria	154	1 (0.6)	13 (2.6)	30 (2.7)	40 (6.9)	23 (6.7)	21 (9.4)	26 (17.2)
Fungal infection	151	9 (5.7)	36 (7.2)	43 (3.9)	22 (3.8)	19 (5.6)	13 (5.8)	9 (6.0)
Bacterial infection	120	11 (7.0)	18 (3.6)	36 (3.3)	26 (4.5)	13 (3.8)	12 (5.4)	4 (2.6)
Protozoal infection	92	5 (3.2)	2 (0.4)	17 (1.5)	27 (4.7)	30 (8.8)	9 (4.0)	2 (1.3)
Keratinization disorders	42	0	14 (2.8)	14 (1.3)	5 (0.9)	3 (0.9)	4 (1.8)	2 (1.3)
All other	220	10 (6.3)	23 (4.6)	47 (4.3)	56 (9.7)	37 (10.8)	27 (12.1)	20 (13.2)

Values are number (percent).

Pigmentary disorders and papulosquamous disorders occured more frequently in winter and spring (12.4% and 5.9%, respectively) as compared to summer and autumn (9.6% and 4.3%, respectively) (**Table 3**). A significantly higher occurrence of protozoal infections was recorded in summer and autumn as compared to winter and spring (4.3% vs. 2%; *P*<.05). Seasonal variation was not significant in other diseases.

**Table 3:** Seasonal variation of skin diseases.

		Season		
Disease	Total	Winter and spring (n=1691)	Summer and autumn (n=1360)	*P*
Eczema/dermatitis	595	333 (19.7)	262 (19.3)	.767
Viral infection	507	289 (17.1)	218 (16.0)	.434
Pilosebaceous disorders	440	227 (13.4)	213 (15.7)	.071
Pigmentary disorders	341	210 (12.4)	131 (9.6)	.015^a^
Hair disorders	231	127 (7.5)	104 (7.6)	.947
Papulosquamous disorders	158	100 (5.9)	58 (4.3)	.041^a^
Urticaria	154	89 (5.3)	65 (4.8)	.497
Fungal infection	151	85 (5.0)	66 (4.8)	.753
Bacterial infection	120	77 (4.6)	43 (3.2)	.053
Protozoal infection	92	34 (2.0)	58 (4.3)	.001^a^
Keratinization disorders	42	25 (1.5)	17 (1.2)	.471
All other	220	95 (5.6)	125 (9.2)	.001^a^

Values are number (percent).

## DISCUSSION

Many epidemiological studies around the world, including a few from Saudi Arabia, have claimed that female patients predominate at dermatology clinics due to the greater sensitivity of women to health-related issues.^6^–^8^ In contrast, our study indicated that male patients attended dermatology clinics more frequently. This finding is also supported by many other studies from Saudi Arabia.^2^,^5^,^9^ The higher prevalence of male patients at dermatology clinics may be attributed to under-representation of females at Saudi clinics due to cultural barriers. Our results also indicated a trend for female patients to outnumber male patients between ages 5 and 24 years. After this age, the number of male patients was greater than that of female patients (Figure 1). This observation indicates that women at younger ages are more conscious of their body image, but lose interest as they grow older. Another explanation for this phenomenon could be the preponderance of female patients with acne presenting around this age (**Table 2**).

The major bulk of our patients (71%) presented between ages 5 and 35 years. The major disease burdens were eczema/dermatitis, pilosebaceous disorders and viral infections, which occurred with greater frequency around this age (**Table 2**). The prevalence of various skin diseases varies according to geographical area and is related to racial, environmental and socioeconomic factors of the population. Eczema and dermatitis are the most prevalent skin disorders reported from developed countries, whereas skin infections are predominant in developing African and Asian countries.^6^,^10^ In a recent study from Tunis, Souissi et al reported 38.6% rate of skin infections in a series of 28 224 patients. Fungal infections were the most frequently seen skin infection (43.8%) in their study.^11^ Similar results have been reported from Iran by Baghestani et al. Infectious and parasitic diseases, including infections of the skin and subcutaneous tissues, were found to be the most common skin diseases (32.1%) in their study.^7^ A review of literature has indicated that bacterial infections tend to decrease in frequency with improved quality of life and better hygienic conditions,^11^ whereas advanced industrialization results in a high prevalence of industrial dermatitis and allergic contact dermatitis.^10^

The overall prevalence of skin infections, including viral, fungal, bacterial and protozoal infections, was 28.5% in our study. Most skin infections were viral. Our results have shown that infections, eczema/dermatitis, pilosebaceous disorders, pigmentary lesions and hair disorders ranked among the top 5 skin diseases (Figure 2). The results of this study are comparable to those of other studies from Riyadh, Abha, Jouf, Hail, Jeddah, Najran, the Eastern Province and the Asir region of Saudi Arabia.^2^,^3^,^5^,^8^,^9^,^12^,^13^ These studies have reported a similar pattern of skin diseases in Saudi patients.

Seasonal variations in certain skin disorders are a well-known phenomenon that has been observed for centuries. A change in temperature, humidity, ultraviolet rays, wind and atmospheric pollen allergens occurs with changing seasons. Low temperature and humidity can have a detrimental effect on the epidermal barrier.^14^ Similarly, warm and humid environmental conditions are conducive to the development of fungal infections.^15^ Hancox et al studied the seasonal variation of dermatological diseases in the USA, and their results confirmed a significant seasonality for skin diseases (*P*=.002). The prevalence of actinic keratosis was significantly higher in spring, while acne and folliculitis were significantly more prevalent in winter. They also noted a trend towards seasonality in cases of dyschromia, seborrheic keratosis, seborrheic dermatitis, and psoriasis. Spring was the peak season for dyschromia, seborrheic keratosis and seborrheic dermatitis, while psoriasis was more prevalent in winter.^16^

When comparing the climate in our study setting with the climate in the settings of the studies conducted in Saudi Arabia, we observed that Riyadh, Jouf, Hail were similar, while the Asir region had a moderate climate and was rainy at most times of the year and had higher humidity levels than Qassim, whereas Jeddah and the eastern region have hot humid summers and moderately cold winters.^17^ The results of our study support some of the observations made in the study by Hancox et al. We found a significantly higher rate of pigmentary disorders and papulosquamous disorders during spring and winter (*P*=.015 and .041, respectively). We hypothesize that the possible reason for an increased prevalence of pigmentary disorders during winter months may be an erroneous belief among people that the role of the sun in affecting the skin is only during the time when the sunrays are hot (summer), so during winter (as the rays are not causing any feeling of hotness on the skin) most people do not follow protective measures to avoid the harmful effects of the sun. Protozoal infections were significantly more frequent in summer and autumn (*P*<.001). Seasonal variation was not significant in other diseases (**Table 3**).

This is the first study to describe the pattern of skin diseases in the Qassim region of Saudi Arabia. Because this study was conducted at Qassim University Medical College-affiliated dermatology clinics of the Ministry of Health, our results provide a rough estimate of the incidence of skin diseases in the Qassim region. However, due to the larger number and homogeneity of our study subjects, we believe that our results may actually mimic the pattern of skin diseases prevalent in the general population of the Qassim area. However, we recommend a large-scale population-based epidemiological study to estimate the true pattern of prevalent skin diseases.

In conclusion, infections, eczema/dermatitis, pilosebaceous disorders, pigmentary lesions and hair disorders ranked among the top five skin diseases. All primary health care physicians and general practitioners should be able to diagnose and manage these common skin disorders. All the cases that were seen in the university-affiliated dermatology clinics of the Ministry of Health were referred from primary health care clinics. We recommend that a series of training programs for diagnosing and managing common skin disorders be initiated for primary health care physicians and other general practitioners. This will result in a decrease in referrals to the dermatology clinics and provide cost-effective and efficient dermatology-related health services at the primary health care level. This study gives us a fair picture of the conditions on which the training of primary health care physicians should be focused. These conditions are skin infections (mainly viral), atopic dermatitis, contact dermatitis, acne vulgaris, vitiligo, post-inflammatory hyperpigmentation, melasma, alopecia areata and diffuse hair loss.
